# Spiritually Integrated Treatment of Depression: A Conceptual Framework

**DOI:** 10.1155/2012/124370

**Published:** 2012-04-19

**Authors:** John R. Peteet

**Affiliations:** Department of Psychosocial Oncology and Palliative Care, Dana-Farber Cancer Institute and Brigham and Women's Hospital, Boston, MA 02115, USA

## Abstract

Many studies have found an inverse correlation between religious/spiritual involvement and depression. Yet several obstacles impede spiritually integrated treatment of depressed individuals. These include specialization and fragmentation of care, inexperience of clinicians and spiritual care providers, ideological bias, boundary and ethical concerns, and the lack of an accepted conceptual framework for integrated treatment. Here I suggest a framework for approaching these obstacles, constructed from a unified view of human experience (having emotional, existential, and spiritual dimensions); spirituality seen as a response to existential concerns (in domains such as identity, hope, meaning/purpose, morality, and autonomy in relation to authority, which are frequently distorted and amplified in depression); a rationale for locating spiritually oriented approaches within a clinician's assessment, formulation, and treatment plan; and recognition of the challenges and potential pitfalls of integrated treatment.

## 1. Introduction

Depressed individuals often feel not only unfortunate but also that the world is oppressive, life is meaningless, and/or God is disapproving. They may question: “Am I clinically depressed, Or lacking in faith?” “Is life as unfair and empty as it seems?” “Is God punishing me?” or “Should I take an antidepressant, or pray more?” Because depression can so closely resemble ordinary spiritual experience, some sufferers resist treatment because they feel they should have more faith. Religious traditions and the communities that interpret them at times regard depression as an illness, at times as evidence of spiritual weakness, and at times even as a punishment.

For their part, mental health professionals may hesitate to address the spiritual dimension of their patients' experience. Some view spirituality as an epiphenomenon of more basic neurobiological or evolutionary processes and as such of only peripheral interest to psychiatry. Others regard religion as a potentially harmful, immature form of wish fulfillment. Still others have ethical concerns about charging patients and/or their insurance companies for spiritually oriented interventions or about influencing patients on the basis of their own personal values. Many lack sufficient familiarity with their patients' spiritual traditions and/or experience to collaborate effectively with religious professionals and/or retain unresolved conflicts in their own relationship with spiritual authorities.

A growing literature describes the spiritual dimension of depression as experienced by its sufferers [1–3], epidemiologic and other evidence for religion as a risk and protective factor [4–6], the social dimension of depression [7, 8], the potential for spiritual growth in the face of adversity [9], ways for depressed individuals to draw upon the resources of a particular faith tradition [10], frameworks for addressing spiritual issues generally in psychotherapy [11–15], and evidence for the effectiveness of spiritual interventions in depression [16–18]. However, the literature has lacked a practical, comprehensive way of approaching the spiritually integrated treatment of depressed individuals of any spiritual tradition or of none.

What follows is a conceptual framework for approaching the complex relationship among depression, spirituality, and mental health treatment, which I more fully describe in *Depression and the Soul: A Guide to Spiritually Integrated Treatment* [19].

## 2. The Spiritual Dimension of Depression

Literature on caring for the whole person recognizes that human suffering includes not only cognitive, emotional, and volitional but also existential and spiritual dimensions. The National Consensus Project Clinical Practice Guidelines for Quality Palliative Care [20] aim to “…identify and address the physical, psychological, spiritual, and practical burdens of illness.” Psychiatrists such as Verhagen [21] have also suggested that the World Health Organization (WHO) recognize spiritual well-being, as an important aspect of health.

However, achieving consensus on definitions of spiritual and existential distress (and by extension care) remains an elusive goal. An integrated literature review of 156 papers dealing with existential suffering in the palliative care setting found 56 definitions [22]. Common themes included the loss of meaning or purpose in life, a sense of connectedness, hope or hopelessness, feelings of loneliness, fear of being a burden to others, a sense of isolation, and an intense fear of dying. But the authors write: “The most prevalent finding in this review has been a lack of consistency in the way existential suffering is defined and understood.” A Consensus Conference on Improving the Quality of Spiritual Care as a Dimension of Palliative Care proposed the following definition: “Spirituality is the aspect of humanity that refers to the way individuals seek and express meaning and purpose and the way they experience their connectedness to the moment, to self, to others, to nature and to the significant or sacred” [23]. Unfortunately, this conception is so broad as to be practically impossible to operationalize.

One important step toward a useable consensus would be to agree on the meaning of terms commonly used to describe spiritual, existential, and emotional distress: as a suggested example, the emotional dimension of patients' concerns could be said to refer to feelings, the existential dimension to the conditions of existence (e.g., in domains such as identity, hope, meaning/purpose, morality, autonomy/connection), and the spiritual to meaningful connections to something larger, transcendent, or sacred [24]. Acceptance of the distinctions drawn by such a vocabulary could help clinicians and researchers identify when particular concerns (such as a sense of isolation, or hopelessness) share, for example, two or three of these dimensions, and are therefore incapable of adequate description by only one. An obvious relationship between spiritual and existential dimensions understood in these terms is that spirituality can function to provide a response to concerns of an existential nature. An additional relevant distinction is that highlighted by the philosopher Charles Taylor between optional, voluntarily embraced ways of finding or investing meaning within an immanent frame such as one might find in nature or art, and experiences on the other hand that lay claim on one because of their ultimate significance, such as one might have with cosmic forces, moral ideals, or God [25].

## 3. Fostering Helpful Spirituality

Consider briefly some of the ways in which a healthy spirituality (in both immanent and ultimate forms) constitutes a helpful response to existential concerns that are frequently amplified and/or distorted in depression, in domains such as identity, hope, meaning/purpose, morality, and autonomy in relation to authority: with respect to identity, a work-oriented businessman who wonders after a heart attack if he is the same person might decide, “this experience has helped me see what I value most.” Or, “I know I am loved, or worthwhile because God loves me.” Coming to such transcendent answers is facilitated by a spirituality that is engaged, and transformative rather than static—whether in relation to the Four Noble Truths of Buddhism or the teaching of Jesus that one must lose one's life to save it.

With respect to hope and its important relational underpinnings, when a loss or a serious illness shakes a religious person's trust in God, he or she can become cynical or despair. Patients who ground their ultimate hopes in ideals such as compassion, truth or justice may also be vulnerable to despair if disillusioned by individuals who have represented these ideals in their lives. Whatever the objects of their faith, patients who have lost hope require a spirituality that is integrated rather than ambivalent or torn. As Judith Herman points out in her book *Trauma and Recovery* [26], a survivor of trauma needs to reconstruct a fragmented view of the world. A hope-sustaining spirituality is one that is accessible and real to the individual not only when he is in a comforting (e.g., a religious) setting but also when he is in the middle of the stress of his everyday life. The theologian Paul Tillich [27] called this the courage to be. Many traditions encourage “spiritual disciplines” (such as prayer, worship, fasting, or giving to others) that help believers to maintain a consistent and coherent connection of their whole selves with their faith.

Many individuals bring into treatment their search for purpose and the larger meaning of their suffering [28, 29]. An atheist who loses a child to cancer may question whether his/her life has any purpose. A religious trauma survivor may question whether s/he can continue to believe that God is fair or loving. Whatever their world views, patients in search of meaning need a spirituality that is contemplative and attuned rather than distracted, impulsive, or self-centered. Both existentialists such as Frankl and Crumbaugh [30] and researchers such as Robert Cloninger et al. [31] have called attention to the central role of self-transcendence in mature personality functioning. Attunement to music, art, or nature as well as prayer and worship can all help one maintain perspective and a center of gravity outside the self. Mindfulness, acceptance, and meditation as means to this end are now taught not only by Buddhist practitioners but also increasingly in psychiatric treatments such as Dialectical Behavior Therapy (DBT), addiction treatment programs, and to patients in general hospital settings.

Patients often present with struggles that have important moral aspects [32]. These are shaped by their world view, in several ways: people's understanding of God and of the universe shapes their commitments to justice, caring, honesty, or community. Philosophical or religious ways of thinking (e.g., depending on versus questioning authority) guide the way people make moral decisions. Religious traditions both articulate standards of right and wrong as well as offer options for dealing with moral failure (e.g., confession, forgiveness, making amends). Faith-based communities and community service organizations help support virtues that are basic to clinical work, such as integrity, equanimity, humility, honesty, and caring. Regardless of differences in their world views, patients with moral concerns need a spirituality that is mature rather than developmentally delayed. Hospital chaplains often refer to the challenge of helping adults who are facing a crisis to call upon a conception of God that goes beyond what they took from Sunday School and is more consonant with their state of emotional maturity. James Fowler in his controversial book *Stages of Faith* [33] pioneered consideration of the ways that faith development, like moral development, is a developmental process. Clinicians can help patients who are otherwise mature to see this and begin to “catch up,” for example, by seeing the advantages of choosing mature connection and intimacy through forgiveness over the more childish satisfactions of maintaining control, or of being “right.” 

The world views of religious and nonreligious individuals tend to differ most sharply on the question of their relationship to an ultimate authority. Is there an authority whom one can trust for care and direction, or does one need to rely on oneself? If God exists, is He an authority who resents His creatures' autonomy, or more like the father in Jesus' parable, more ready to receive the prodigal son home than the son imagines? Whatever one's world view, there are benefits, as Pargament's research has shown [34], to feeling loved rather than rejected by the Other. Clinicians can help patients to look at what kind of intimacy with God and others is possible. Is there a community that is more welcoming of the patient than he can see? Interpersonal therapeutic approaches and attention to the ways that spiritual communities address the dynamics of relationships with others and the Other are particularly apt here.

## 4. Spiritually Integrated Treatment

What is the place of spiritually oriented approaches within a clinician's assessment, formulation, and treatment plan? Table 1 outlines a general framework for intervening at the interfaces between emotional, existential, and spiritual distress in the domains of depressed individuals' core concerns, to foster a more healthy spirituality. Whereas insight-oriented and cognitive behavioral approaches can help depressed individuals to distinguish distressing emotions from their actual basis in life experience, spiritually oriented interventions can help them use their knowledge and experience of their spirituality (in its ultimate sense, where God or morality are involved) to put these experiences into a larger perspective.

In 1973, Akiskal and McKinney amassed a large body of evidence in support of a unitary hypothesis according to which the depressive syndrome is a “psychobiological final pathway” [35, page 286]. Its symptoms are familiar: persistent feelings of sadness, difficulty concentrating, indecisiveness, hopelessness, pessimism, guilt or worthlessness, fatigue, lack of energy and initiative, an impaired capacity for enjoyment, disturbances of sleep and appetite, and thoughts of death or suicide. Their conception came to dominate the field and shaped the category of Major Depressive Disorder in the Diagnostic and Statistical Manual (DSM). It also fits popular descriptions of depression by sufferers such as Solomon (*The Noonday Demon: An Atlas of Depression* [2]) and Styron (*Darkness Visible: A Memoir of Madness* [3]), whose individual illnesses seemed to take on a life of their own, eventually depriving them of rational perspective and a sense of control.

However, a number of investigators have questioned whether this model may be overly simplistic. Parker [36] has suggested that depression without psychomotor (melancholic) or psychotic features is better regarded as a spectrum of disordered responses to life that are “induced and/or maintained by predisposing factors” (page 1199). Kendler et al. [37] have similarly proposed a model of major depression that is etiologically diverse, “influenced by risk factors from multiple domains that act in developmental time” (page 115). Horowitz and Wakefield [38] go further to suggest that psychiatry's system of classification has “transformed normal sorrow into depressive disorder.” And from a therapeutic point of view, Schatzberg [39] has called on clinicians to move beyond symptom control to manage the underlying vulnerabilities that contribute to recurrent depression.

Viewed from a stress diathesis perspective, it seems clear that several conditions confer a vulnerability to a depressed mood and that they differ in their etiologies as well as in their therapeutic implications. These include melancholia, demoralization, bipolar disorder, adjustment disorder, personality-related depression, angst, addiction-related depression, guilt, trauma-related depression, the “Dark night of the soul”, complicated grief, and ordinary unhappiness. Obviously, the core concerns (e.g., mistrust and shame following trauma, perfectionism, a negative self-identity and self-sacrifice) of individuals with these conditions and their existential and spiritual dimensions are likely to differ.

Table 2 suggests ways that specific spiritually informed interventions can address the existential dimension of depressive concerns. For example, patients whose existential concerns center around identity, and who are therefore vulnerable to experiencing doubt or disorientation when depressed, may benefit from a humanistic emphasis on connecting with what most fulfills and best defines them. If religious, they may also benefit from grounding their identity in their relationship to God, for example, through a process of spiritual direction.

Patients with difficulty maintaining ultimate hope because their experience of the world is fragmented, and who are mistrustful when in despair, would be expected to benefit from achieving a more integrated spirituality through, for example, exploration of unresolved trauma, CBT that brings their core beliefs more in line with their experience, and interpersonal therapy or spiritual direction that focuses on their doubts about trusting God, or the future.

Depressed patients who struggle to find a sense of meaning, or who feel their life has lost its purpose would be expected to benefit from meaning-centered therapy, mindfulness, and meditation.

Patients concerned with moral questions, such as those who feel overwhelmed by guilt when depressed, would be expected to benefit from forgiveness promoting therapy and the emphasis of positive psychology on virtues such as love.

Patients whose existential concerns center on their relationship to ultimate authority, and who feel isolated or rejected when depressed, would be expected to benefit from feeling accepted and loved by God. Potential therapeutic means to this end include psychodynamically oriented treatment focused on their distorted object relations, interpersonal therapy focused on their relationship to God, and/or spiritual direction.

Spiritually oriented approaches that address concerns in one of these domains—for example, one's relationship to God—may of course also address concerns in other areas, such as identity or hope. For example, the individual who feels loved by God, worshipful, and continually surrendered to his will may be less prone to worship lesser gods such as power or pleasure that will disappoint and leave him depressed.

There are a number of venues in which integrated treatment can be provided, ranging from the office of a clinician in a secular office or hospital, to that of a religiously committed therapist in a faith-based clinic, to that of a pastoral counselor in a church. Each presents its own challenges and opportunities for collaboration, referral, and sharing of expertise [19, (see pages 169–184)]. Elsewhere, I have distinguished four possible roles of a psychotherapist in approaching spiritual problems (such as a crisis of faith, paralyzing guilt, or religious objections to taking medication) [40].

In the most familiar and straightforward of these, a therapist would acknowledge the problem, but limit discussion to its psychological (or strictly medical) dimension. For example, he might focus on how the problem is interfering with the patient's care or address a patient's anger at God by examining his relationship with other authority figures in the patient's life.

A second possible approach would be to clarify the spiritual as well as the psychological aspects of the problem, suggest resources for dealing with the former, and consider working with an outside resource such as a religious community or other authority. This might include enlisting a hospital chaplain or clergy person to offer needed spiritual help or referring a patient to a therapist of a similar tradition. It could also include referral to organized programs that integrate beliefs and emotions, such as religiously/spiritually based cognitive behavioral or Twelve-Step programs.

In a third approach, a therapist would aim to address the problem indirectly using the patient's own philosophy of life within the treatment. This might include exploring ways the patient can make better use of his resources and tradition (e.g., by examining a range of beliefs within the patient's own denomination, or misconceptions about the spiritual nature of AA). Here it is helpful for therapists to appreciate how different world views and spiritual traditions address existential concerns, such as identity. For example, in the Judeo-Christian tradition each individual is contingent (as created), broken (sinful) and in need of healing (forgiveness and transformation), and loved unconditionally; in the Buddhist tradition, each individual is at one with the universe, unhappy but capable of self-emptying and of enlightenment; in a secular Western view, each individual is limited by bias but evolving, ultimately alone but capable of living with integrity.

A fourth approach would be to address the problem directly together using a shared perspective, ranging from the therapist's agreement on the importance of hope, meaning, world view, or a caring community to the prescriptive use of shared values, beliefs, or practices (e.g., meditation or scripture) in the treatment. This fourth approach requires particularly careful attention to transference, countertransference, boundary, and consent issues.

A number of factors are relevant in deciding which of these approaches to take. The first is the patient's need—whether for growth, adjustment, or problem solving. This in turn influences the nature, primary aims, and timing of the work—for example, psychological insight into a maladaptive pattern or resolution of a conflict. These in turn influence the degree of direct support needed and the amount of interpersonal closeness that is appropriate. Additional factors include the patient's existential concerns—for example, related to hope or identity—and the spiritual options under consideration, the importance of spirituality in his life, his presenting problem and attitude toward treatment, the concern of the patient to integrate psychological with other perspectives; the availability of outside philosophically or spiritual resources, and the therapist's own knowledge and preferred style. Dual relationships, for example, being a treater as well as a fellow member of the same religious community, complicate the transference, countertransference, and boundary aspects of taking one or another approach. 

## 5. Remaining Challenges

The framework for integrated treatment suggested here raises a number of challenging questions: Which aspect of a depressed individual's condition should have priority, and which should receive a spiritually oriented approach? What is the relationship of the important biological and genetic components of serious depression to its spiritual dimension [41]? What is the importance of the patient's and the clinician's world view in formulating the goals of spiritual care? What boundaries are important to maintain in dealing with religious and spiritual issues, for example, regarding disclosure of the therapist's own world view? What are the pitfalls of either neglecting or overemphasizing spirituality? Partial answers are emerging from the literature on addressing spiritual issues generally in psychotherapy [42]. Fuller answers, still emerging from work with patients struggling with depressive concerns, are needed to elucidate more clearly the mechanism of action of spiritually oriented interventions and to establish best practices in providing integrated, whole person care.

## 6. Conclusion

Interest continues to grow in understanding the place of spirituality in depression, but consensus has been difficult to achieve about how best to approach the intertwined emotional, existential, and spiritual dimensions of patients' depressive concerns. The framework suggested here emphasizes the need for clinicians to consider a broad range of diagnostic categories and dynamic concerns arising in depressive conditions, to recognize the existential dimension of these concerns in areas such as identity and hope that are causing emotional distress, to identify corresponding goals for an appropriately helpful spirituality, and to select interventions accordingly, so as to provide individualized, comprehensive treatment.

## Figures and Tables

**Table 1:** A framework for intervention.

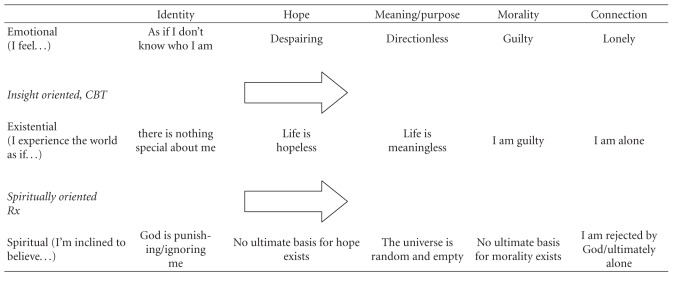

**Table 2:** The relationship of spiritually oriented interventions to depressive concerns.

Existential/clinical domain	Depressive concern	Healthy spiritual characteristic	Spiritually oriented approach
Identity	Doubt, disorientation	Engaged	Humanistic, 12 Step
		Transformative	
Hope	Despair, mistrust	Integrated	Psychodynamic,
			CBT
		Visionary	Spiritual direction, IPT
Meaning/purpose	Meaninglessness	Attuned, contemplative	Meaning centered, mindfulness, meditation
Morality	Guilt	Mature, reconciled	Forgiveness promoting, positive psychology
Authority/autonomy	Isolation, rejection	Accepted, loved	Psychodynamic, IPT, spiritual direction
